# Dextran Conjugation Improves the Structural and Functional Properties of Heat-Treated Protein Isolate from *Cinnamomum camphora* Seed Kernel

**DOI:** 10.3390/foods11193066

**Published:** 2022-10-02

**Authors:** Xianghui Yan, Xiaofeng Gong, Zheling Zeng, Maomao Ma, Junxin Zhao, Jiaheng Xia, Meina Li, Yujing Yang, Ping Yu, Deming Gong, Dongman Wan

**Affiliations:** 1 State Key Laboratory of Food Science and Technology, Nanchang University, Nanchang 330047, China; 2 Jiangxi Province Key Laboratory of Edible and Medicinal Resources Exploitation, Nanchang University, Nanchang 330031, China; 3 School of Resources and Environment, Nanchang University, Nanchang 330031, China; 4 School of Chemistry and Chemical Engineering, Nanchang University, Nanchang 330031, China; 5 School of Food Science and Technology, Nanchang University, Nanchang 330031, China; 6 New Zealand Institute of Natural Medicine Research, 8 Ha Crescent, Auckland 2104, New Zealand

**Keywords:** plant protein, Maillard reaction, conjugate, structural property, functional property

## Abstract

The *Cinnamomum camphora* seed kernel (CCSK), with high contents of medium-chain oil (~59%) and protein (~19%), is an excellent source for a plant-based food ingredient. To broaden the application of the protein isolate (PI) from CCSK in the food industry, the Maillard reaction products (MRPs) were prepared by PI and dextran (DX) under mild wet-heating conditions (60 °C, 5 h), and the structural and functional properties of the PI-DX conjugates were investigated. The covalent bond between PI and DX was confirmed by the degree of grafting and sodium dodecyl sulfate-polyacrylamide gel electrophoresis. Compared with the heated PI, the PI-DX conjugates had more ordered structure, with the decreased random coil content. The changes in tertiary structure of PI-DX conjugates were reflected by the results of intrinsic fluorescence and surface hydrophobicity. Moreover, PI-DX conjugates showed better solubility, emulsifying properties, thermal stability and antioxidant activities. These results provided a theoretical basis for the development of PI-based MRPs with desirable characteristics.

## 1. Introduction

Plant-based proteins have received increasing attention because of their sustainability and nutritional values. The *Cinnamomum camphora* seed kernel (CCSK) is a natural source of plant-based medium carbon chain triglycerides and proteins, with the contents of 59% and 19%, respectively [1]. As a massive broad leaved evergreen, the *Cinnamomum camphora* (L.) Presl tree is widely distributed in subtropical and tropical Asia. In particular, it is widely cultivated as a type of landscape tree in the southern part of the Yangtze River in China, with the annual production of *Cinnamomum camphora* seeds exceeding 1 million tons. As a by-product of oil production, the defatted meal from CCSK contains around 49% protein, as well as other valuable components, such as dietary fiber, phenolic compounds and minerals [1,2]. The content of essential amino acids in CCSK protein meets the recommendation of the Food and Agriculture Organization (FAO) and the World Health Organization (WHO) for adults [1]. However, similar to most of the plant proteins [3,4], the application of CCSK protein is greatly limited in the food industry due to its inferior functional properties and susceptibility to pH value, ionic strength and temperature [5]. Therefore, the modification of PI to improve its functional properties could be an alternative approach to making their applications in foods more feasible.

Different modification methods, including physical, chemical, enzymatic and complexation, were used for the modulation of plant proteins [6]. Among them, the protein glycosylation induced by the Maillard reaction is widely used to broaden the functionality of plant proteins [7,8,9]. During the Maillard reaction, irreversible covalent bonds between the free amino groups of protein and the carbonyl groups of reducing sugar are formed. As an excellent modification method, the reaction can not only combine the original properties of polysaccharides (e.g., solubility and emulsifying properties) but also create new properties of proteins, especially antioxidant activity and flavor attributes [10,11,12]. However, the production of the Maillard reaction products (MRPs) is mainly affected by reaction temperature, time, pH value, humidity and mass ratio of the reactants [13]. Usually the Maillard reaction process is accompanied by heat treatment of proteins. The soluble/insoluble protein aggregates are likely to be formed at high temperatures, which may result in the irreversible loss of functional properties in MRPs. In addition, the extent of the Maillard reaction may also be affected by the formation of protein aggregation at high temperatures [14,15]. From the point of view of industrial production, it is desirable to obtain MRPs under mild reaction conditions with good functional properties. A study by Li et al. [10] found that the emulsification and foaming properties of soy PI were significantly improved by conjugating with glucose at 60 °C.

Currently, there is limited information available on the structural characteristics and functionalities of PI glycosylation. This study aimed to prepare PI–dextran conjugates under mild wet-heating conditions. The role of dextran conjugation on the secondary and tertiary structures of MRPs was analyzed. The solubility, emulsifying properties, differential scanning calorimetry and antioxidant activities were investigated to evaluate the functional properties of MRPs.

## 2. Materials and Methods

### 2.1. Materials

The defatted *Cinnamomum camphora* seed kernel (DCCSK, protein ~50%, lipid ~6%, moisture ~4%) was prepared by our laboratory. Dextran (DX, >99%) with a molecular weight of 40 kDa was purchased from Aladdin Biochemical Technology Co., Ltd. (Shanghai, China). o-phthalaldehyde (OPA), 8-anilino-1-naphthalenesulfonic acid (ANS), sodium dodecyl sulphate and β-mercaptoethanol were purchased from Solarbio Technology Co., Ltd. (Beijing, China). The Bradford Protein Assay Kit from Shandong Sparkjade Biotechnology Co., Ltd. ddH_2_O (Milli-Q, Beijing, China) was used to prepare solutions in the experiments.

### 2.2. Preparation of Protein Isolate (PI) from DCCSK

#### 2.2.1. Extraction of Polyphenol

Polyphenol was extracted from DCCSK according to our previous study [1]. Briefly, the DCCSK flour was dispersed in 80% (*v*/*v*) ethanol-aqueous solution at a ratio of 1:20 (*w*/*v*) with stirring at room temperature for 2 h, and the extraction process was performed five times. The total phenolic content in DCCSK before and after the removing were 21.20 and 0.15 mg gallic acid equivalent/g. The remaining residue was obtained for the extraction of PI.

#### 2.2.2. Extraction of PI

PI was extracted from DCCSK flour free of polyphenol based on the traditional alkaline/isoelectric precipitation extraction method described by Guimarães Drummond and Silva et al. [16]. Briefly, DCCSK flour was mixed with ddH_2_O at a ratio of 1:15 (*w*/*v*) and the pH value of dispersion was adjusted to 9.0. The dispersion was stirred at 25 °C for 1 h and then centrifuged at 3500 rpm for 20 min (LXJ-IIB, Shanghai Anke Scientific Instrument Factory, Shanghai, China). After repeating the extraction process once again, the supernatants containing proteins were collected. The supernatant was then precipitated at a pH value of 4.0 and centrifuged at 3500 rpm for 10 min. The protein precipitate was suspended in ddH_2_O, and the pH value was adjusted to 7.0. The insoluble fraction was removed by centrifugation at 3500 rpm for 10 min, and the supernatant was dialyzed, lyophilized and stored at −20 °C for further use.

### 2.3. Preparation of PI-DX Conjugates

The PI-DX conjugates were prepared according to Li et al. [10] with some modifications. Briefly, freeze-dried PI was mixed with DX flour at a mass ratio of 2:1, 1:1 and 1:2 (*w*/*w*), respectively. The mixed flours were dispersed in phosphate buffer solutions (10 mM, pH 7.0), to ensure the PI concentration was 2% (*w*/*v*). The dispersions were then stirred at 200 rpm at 25 °C for 2 h and stored at 4 °C overnight for completely hydration. Then, the solutions were heated in a thermostat water bath at 60 °C for 5 h and then promptly cooled in an ice-water bath. After freeze-drying, the MRPs of PI-DX conjugates were obtained. The PI heated alone was used as a control.

### 2.4. Measurement of UV Absorbance

UV absorbance values of the samples were measured using a TU-1950 UV-vis spectrophotometer (Purkinje General Instrument Co., Beijing, China) according to the method of Pirestani et al. [17]. The absorbance values of sample solutions were recorded at 294 and 420 nm with protein concentrations of 1.0 and 10.0 mg/mL, respectively. Moreover, the UV absorption spectra were obtained by scanning from 200 nm to 400 nm. The protein concentration of each sample was diluted to 1.0 mg/mL.

### 2.5. Determination of Degree of Grafting (DG)

The DG was measured by the analysis of free amino groups (FAGs) using the o-phthalaldehyde (OPA) spectrophotometric assay [10]. The OPA reagent was freshly prepared from the following reagents combined and adjusted to 100 mL with ddH_2_O: 80 mg of OPA (dissolved in 2 mL of methanol); 5 mL of 20% sodium dodecyl sulphate; 200 µL of β-mercaptoethanol and 50 mL of 0.1 M sodium tetraborate. Aliquots of diluted samples (200 µL) were mixed with 4 mL OPA reagent. The mixture was incubated at 37 °C for 2 min. The UV absorbance value at 340 nm of the mixture was recorded. A standard curve was obtained with L-leucine. The formula for calculating DG was as follows:

DG (%) = [(A_0_ − A_1_)/A_0_] × 100% (1)

where A_0_ is the content of FAGs in PI, and A_1_ is the content of FAGs in PI-DX conjugates.

### 2.6. Sodium Dodecyl Sulfate-Polyacrylamide Gel Electrophoresis (SDS-PAGE)

SDS-PAGE of the samples under non-reducing and reducing conditions was performed, using 12% acrylamide separating gel and 5% acrylamide stacking gel [1].

### 2.7. Determination of Z-Average Size and Zeta Potential

Protein samples were dissolved in ddH_2_O at 1 mg/mL. The particle size and zeta potential of the samples were measured by using a Zetasizer 2000 (Nano-ZS, Malvern Instruments, Worcestershire, UK). The refractive indexes for the protein particles and dispersion medium were set as 1.46 and 1.33, respectively [1].

### 2.8. Structural Characterization of PI-DX Conjugates

#### 2.8.1. Fourier Transform Infrared (FT-IR) Spectroscopy

FTIR spectroscopy of samples was conducted with a Nicolet iS10 spectrometer (Thermo Nicolet Co., WI, USA). Freeze-dried samples were mixed with KBr at a ratio of 1:100 (*w*/*w*) and the spectra of the samples were recorded at wavelength of 4000–400 cm^−1^. The secondary structure contents of samples were calculated according to the method described previously [1].

#### 2.8.2. Intrinsic Fluorescence Spectroscopy

The intrinsic fluorescence spectra of samples were measured by a F-7000 fluorescence spectrophotometer (Hitachi, Tokyo, Japan). Protein samples were dissolved in ddH_2_O at 1 mg/mL. Under the excitation wavelength of 280 nm, the emission wavelengths between 290 and 500 nm were recorded [18].

#### 2.8.3. Surface Hydrophobicity (H_0_)

The H_0_ was measured using ANS as a fluorescence probe according to the method of Wang et al. [19] with minor modifications. Briefly, 1 µL of 8 mmol/L ANS solutions was added to 200 µL of sample solutions at the protein concentrations of 0.05–0.25 mg/mL. The fluorescence intensity was recorded by using a Microplate Reader (Varioskan LUX, ThermoFisher Scientific, Waltham, MA, USA) with the excitation wavelength of 390 nm and emission wavelength of 470 nm. The H_0_ was obtained from the initial slope of the fluorescence intensity plotted against the protein concentration.

#### 2.8.4. Scanning Electron Microscopy (SEM)

The surface structure of samples was observed in an electron accelerating voltage of 5 kV using a scanning electron microscope (Hitachi SU8020, Tokyo, Japan). The powder sample was placed on the conductive adhesive and coated with gold. The characterization results were supported by Beijing Zhongkebaice Technology Service Co., Ltd. (www.zkbaice.cn) and accessed on 24 November 2021.

### 2.9. Functional Properties of PI-DX Conjugates

#### 2.9.1. Protein Solubility and Emulsifying Properties

The protein solubility and emulsifying properties, including emulsifying activity index (EAI) and emulsion stability index (ESI), were determined following our previous study [1].

#### 2.9.2. Differential Scanning Calorimetry (DSC)

The thermal behavior of the samples was analyzed by a DSC250 (TA Instrument, New Castle, DE, USA). The samples (3~6 mg) were accurately weighed and heated from 25 to 200 °C at a rate of 10 °C/min [20]. The variations of heat flow with temperature were recorded.

#### 2.9.3. Antioxidant Activity

The DPPH and ABTS radical scavenging activities of the samples were determined as described by our previous study [21]. For both assays, the protein concentration was adjusted to 1 mg/mL.

### 2.10. Statistical Analysis

Results were reported as means ± SD and measured in triplicate. Data were analyzed by one-way ANOVA, followed by Tukey’s test using SPSS software (SPSS Inc., Chicago, IL, USA). *p* < 0.05 represents the significant difference.

## 3. Results and Discussion

### 3.1. Formation of PI-DX Conjugates

**Figure 1:**
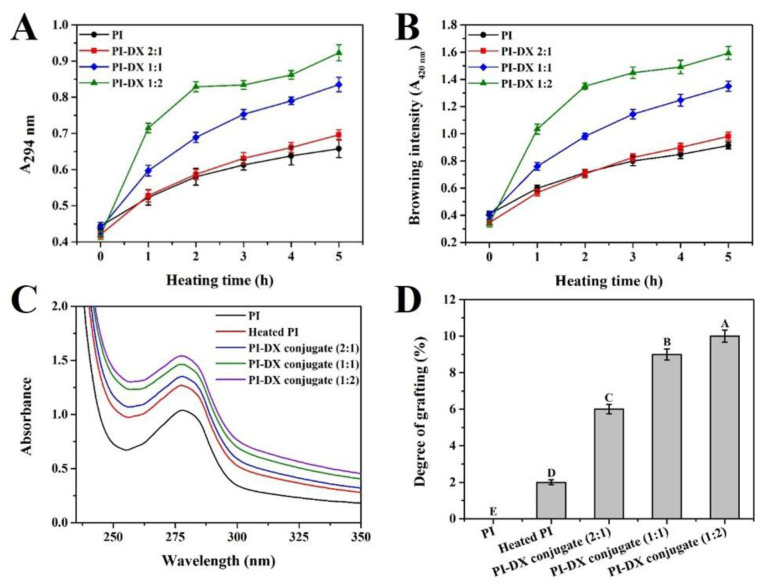
Changes in intermediate products (A_294nm_) (**A**), Browning intensity (A_420nm_) (**B**), UV spectra (**C**), and degree of grafting (**D**) of PI-DX conjugates. PI: protein isolate; DX: dextran. Values with different letters (A, B, C, D, E) in each sample were significantly different (*p* < 0.05).

During the Maillard reaction, the formation of intermediate products, such as the Amadori compounds, at the early stage had characteristic UV absorption at 294 nm (A_294_) and the advanced stage products, such as melanoidins, were determined by measuring the absorbance value at 420 nm (A_420_) [19]. As shown in Figure 1A,B, both A_294_ and A_420_ of the PI-DX conjugates were increased continuously with the increase in reaction time. After 5 h of heating, the PI-DX 1:2 conjugate showed the highest increase in A_294_ and A_420_, followed by those of the PI-DX 1:1 and PI-DX 2:1 conjugates. These results indicated that the Maillard reaction products, Amadori compounds and melanoidins, were produced after the reaction between PI and DX, and the reaction accelerated with the increase in the DX concentration. A strong relationship between A_294_ and A_420_ was also observed in the canola PI–gum Arabic and ovalbumin–dextran Maillard reaction systems [17,22].

The UV absorption spectra of the samples were determined (Figure 1C). The maximum absorbance value of PI was observed at approximately 280 nm, which was characteristic of proteins containing tryptophan, tyrosine, and phenylalanine [23]. The UV absorption of the heated PI was higher than that of native PI. This phenomenon may be because the molecules of PI were unfolded after heating, resulting in increased exposure of the free amino groups in the side chains [24]. For PI-DX conjugates, the increased UV absorption indicated the formation of Schiff bases [25]. Moreover, the UV absorption intensity of PI-DX conjugates was increased with the increase in DX concentration, which also reflected the extent of glycation.

In the primary stage of the Maillard reaction, the covalent attachment between active amino groups and carbonyl groups is mainly to form Schiff base [10]. Thus the DG between protein and polysaccharide could be quantified by the free amino group content. As shown in Figure 1D, compared with the heated PI, the DG values of PI-DX conjugates were significantly increased as DX concentrations were increased. Approximately 3.85, 7.30 and 8.33% of the free amino groups of PI were conjugated with DX for PI-DX 2:1, PI-DX 1:1 and PI-DX 1:2 conjugates, respectively. This result may be due to the high concentration of DX that can increase the chance of covalent attachment of active amino groups to carbonyl groups. A similar result was reported by Shi et al. [26], who found that the Maillard reaction product of whey PI and DX in a weight ratio of 1:2 (*w*/*w*) had the highest DG value, followed by whey PI-DX 1:1 and whey PI-DX 3:1 conjugates.

### 3.2. SDS-PAGE

**Figure 2:**
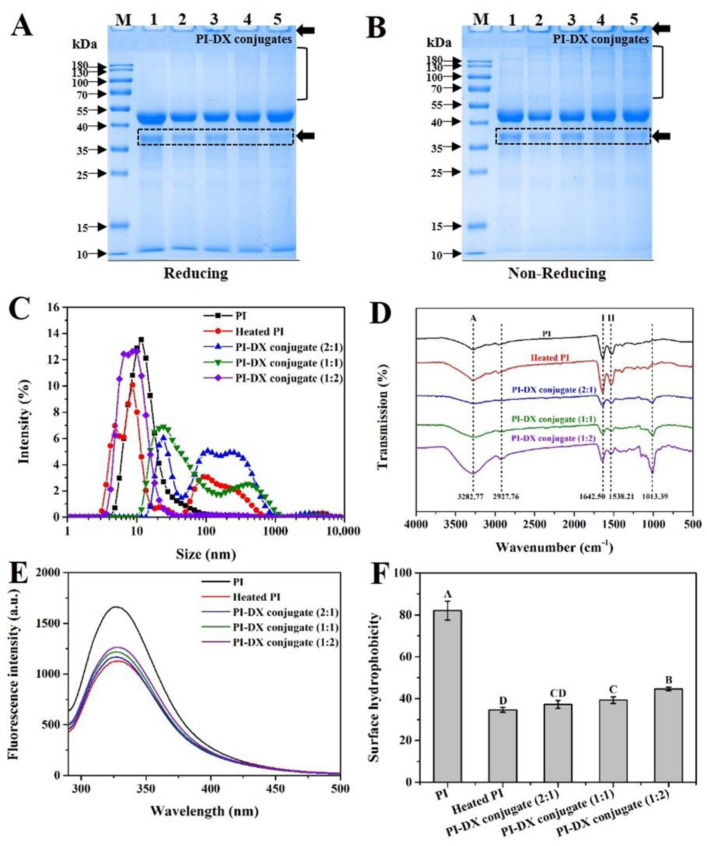
The SDS-PAGE profiles (**A**: reducing; **B**: non-reducing), size distribution (**C**), Fourier transform infrared spectroscopy (**D**), intrinsic fluorescence spectra (**E**), and surface hydrophobicity (**F**) of PI-DX conjugates. PI: protein isolate; DX: dextran. Values with different letters (A, B, C, D) in each sample were significantly different (*p* < 0.05).

The formation of PI-DX conjugates was further characterized by SDS-PAGE. As shown in Figure 2A,B, the characteristic bands at ~40–55 kDa and ~35–40 kDa in PI (Lane 1) were observed both under reducing and non-reducing conditions. These two bands were the major fractions of CCSK PI, consistent with our previous study [21]. No significant difference was observed between PI and heated PI (Lane 2), indicating that the protein primary structure was thermally stable at 60 °C. However, compared with the heated PI, the band intensity of ~35–40 kDa (see the dotted box in Figure 2A,B) was slightly decreased after the conjugation with DX, and new bands with high molecular weights (>55 kDa) were formed. These results revealed that the band at ~35–40 kDa of PI was mainly involved in Maillard reactions with DX. A newly formed visible band near the loading end of the gel was observed in PI-DX conjugates (Lanes 3–5), and these phenomena became more obvious with the increase in DX concentration either under reducing or non-reducing conditions. Therefore, these results further confirmed the covalent link between the carbonyl groups of DX and the free amino groups of PI and that the conjugates with high molecular weights were developed. This finding is consistent with Zhong et al. [8], who observed a characteristic band on the top of the stacking gel by the formation of oat PI—*Pleurotus ostreatus* β-glucan conjugate.

### 3.3. Z-Average Size and Zeta Potential

The particle size is an indicator of changes in protein structure and has an important effect on the functional properties of proteins. As shown in Figure 2C and Table 1, PI exhibited a main peak in the size distribution between 1 and 1000 nm. Heat treatment and DX conjugation induced a shift of the peak distributions towards a bigger size. Specifically, compared to PI (66.33 ± 1.07 nm), heat treatment significantly (*p* < 0.05) increased Z-average size with a value of 142.90 ± 1.65 nm for heated PI. This result suggested that PI may participate in the formation of heat-induced protein aggregates with high molecular weights, which was also reflected by the result of the SDS-PAGE profiles (Figure 2A,B). On the other hand, a slight increase in Z-average size was observed for PI-DX 2:1 (152.90 ± 2.61 nm) when compared to that of heated PI. Interestingly, as the mass ratio of DX in the Maillard reaction system increased, the Z-average size of PI-DX 1:1 and PI-DX 1:2 conjugates decreased significantly (45.53 ± 0.55 and 124.07 ± 1.50 nm, respectively). The effect may be because DX groups increase the steric repulsion between PI molecules, thereby reducing their tendency to aggregate with each other [27].

The zeta potential is an important physical property of proteins that explains the nature of the electrostatic interactions around the surface of the protein molecules [28]. As shown in Table 1, the absolute zeta potential value of heated PI (−23.13 ± 0.61 mV) was significantly higher than that of PI (−21.40 ± 0.70 mV). This result means that heated PI has greater electrostatic repulsion interactions than PI, indicating that heated PI is more stable than PI in the solution. For the Maillard reaction system, the absolute zeta potential value of the PI-DX conjugates was first increased and then decreased as the increase in DX concentration. The highest absolute zeta potential value was found for PI-DX 1:1 conjugate (−24.63 ± 0.46 mV). This may be because the appropriate ratios of DX (PI:DX = 2:1 and 1:1) participating in the Maillard reaction exposed the internal charged groups through unfolding of the protein structure, but the increase in the DX concentration (PI:DX = 1:2) provides the shielding effect of the protein surface charge away from the aqueous phase [29].

### 3.4. Structural Properties of PI and PI-DX Conjugates

#### 3.4.1. FTIR Spectroscopy

The FTIR spectroscopy is widely used to verify the protein–polysaccharides interactions and assess the structural changes of the glycosylated proteins. As shown in Figure 2D, PI exhibited two characteristic absorption bands at amide I (1600–1690 cm^−1^) and amide II (1480–1575 cm^−1^), respectively, which were the most distinctive spectral features for proteins. Generally, the band at amide I mainly refers to C=O stretching vibration, and the band at amide II mainly refers to C–N stretching vibration and N–H bending [30]. For DX, a series of overlapping peaks located in the 910–1150 cm^−1^ region were observed (Figure S1), referred to as the “saccharide” band, mainly due to the C–C and C–O stretching vibrations and C–H bending [31]. Moreover, the broad absorption bands of PI and DX at 3100–3600 cm^−1^ were mainly due to the O–H stretching vibration [32].

The band positions of PI and heated PI did not change significantly (Figure 2D), which is similar to the result of Zhong et al. [8]. After conjugation with DX, the absorption band intensities for all PI-DX conjugates at amide I and II were reduced as compared to heated PI, indicating that the C=O and C-N stretching from amide I and II were modified by the Maillard reaction [31]. The similar phenomenon was also observed for soy PI–lentinan conjugates [20] and ovalbumin–DX conjugates [22]. Meanwhile, as expected, the absorption band intensity of PI-DX conjugates in the region of 910–1150 cm^−1^ gradually increased with the increase in DX concentration, suggesting that covalent bonds were formed between PI and DX. Li et al. [33] also reported a characteristic band at 1015 cm^−1^ in the soy peptide–DX conjugates. The content of secondary structure deduced from the wavelength region of 1600–1700 cm^−1^ (Table 2) was obtained by the fitting of Gaussian peaks (Figure S2). Compared with PI, heat treatment resulted in decreased α-helix and β-sheet contents and increased β-turn and random coil contents, indicating that the protein unfolding occurred upon heating. For the Maillard reaction system, on the contrary, the PI-DX conjugates had lower contents of β-turn and random coil than heated PI, and gradually decreased with the increase in DX concentration. This observation indicated that the secondary structure of PI-DX conjugates became more ordered, possibly because covalently binding with DX increased the intermolecular interaction between protein molecules [34]. Therefore, these results revealed that the secondary structure of heated PI was improved by conjugating with DX, which was consistent with the finding of Zhong et al. [8].

#### 3.4.2. Intrinsic Fluorescence Spectroscopy

The fluorescence spectrum of proteins is mainly related to tryptophan (Trp) residues, which are sensitive to the microenvironment and can be used to reflect changes in the tertiary structure of proteins [35]. As shown in Figure 2E, the fluorescence intensity of heated PI was significantly lower than that of PI. This result may be because heat treatment promotes the formation of disulfide bonds within or between protein molecules, which can be stacked on an internal indole ring, leading to quenching reactions [36]. Figure 2E also shows that the Maillard reaction resulted in enhanced fluorescence intensity of PI-DX conjugates, and the fluorescence intensity increased gradually as a function of DX concentration. The structure of protein–polysaccharide conjugates was reported to be related to the fluorescence intensity [20,35]. The Maillard reaction may be involved in the formation of fluorescent compounds that may be precursors of brown pigments [37]. The higher fluorescence intensity of PI-DX conjugates may be related to the production of more fluorescent compounds. Therefore, it could be inferred that the tertiary structure of PI-DX conjugates was changed, and DX with higher proportions (PI:DX = 1:2) significantly promoted the Maillard reaction.

#### 3.4.3. Surface Hydrophobicity (H_0_)

The H_0_ refers to the number of hydrophobic groups exposed on the protein surface in the dispersion phase, which plays an important role in the functionalities of proteins [38]. Compared with PI, the decrease in the H_0_ value for heated PI was observed (Figure 2F). The H_0_ value of PI was 82.01 ± 4.50 but decreased significantly (34.62 ± 1.17). This may be because heat treatment promotes the protein–protein aggregation, leading to a reduction in the surface area of hydrophobic groups, which was consistent with the result of intrinsic fluorescence spectroscopy. In addition, the H_0_ values of PI-DX conjugates were higher than that of heated PI and gradually increased with increasing DX concentration from PI:DX = 2:1 to PI:DX = 1:2. This phenomenon could be attributed to the exposure of hydrophobic groups buried inside the protein molecules as a result of the attachment of DX to PI under wet-heat treatment [39]. Conversely, it was reported that the H_0_ values of oat PI and soy β-conglycinin decreased after the Maillard reaction in comparison with heating alone [8,15]. This difference may result from different proteins used.

#### 3.4.4. Scanning Electron Microscopy (SEM)

**Figure 3:**
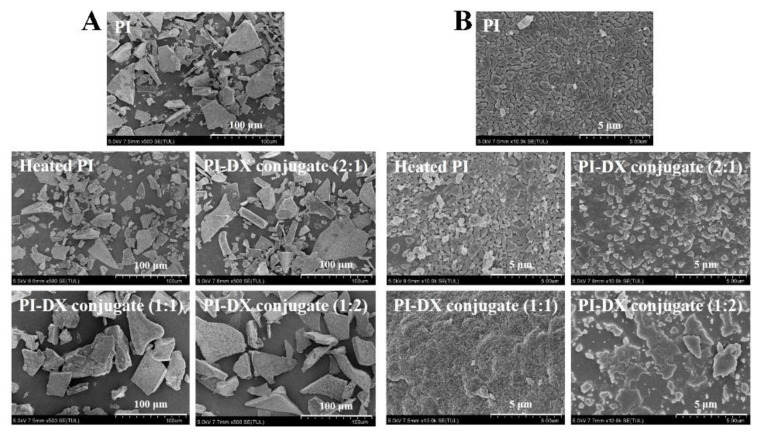
The scanning electron micrographs of PI-DX conjugates (**A**: ×500; **B**: ×10,000). PI: protein isolate; DX: dextran.

Both PI and PI-DX conjugates exhibited a sheet-like structure as typical freeze-dried samples (Figure 3). The number of small fragments in the heated PI was higher than that in PI (Figure 3A). This result was consistent with the structural changes of PI, as confirmed by the result of FTIR. After the Maillard reaction, the structures of PI-DX conjugates became more lumped, which may be because glycosylation could retrench the structure of proteins. As shown in Figure 3B, the surface morphology of PI samples changed significantly after heat treatment and the Maillard reaction, indicating the changed conformation of PI. Compared with PI, the surface morphology of heated PI became looser and more porous. For Maillard reaction products, PI-DX conjugates showed more continuous surface than heated PI, indicating that PI was closely attached to the DX molecule [40]. Similarly, Wang et al. [41] reported that the gel surface morphology became more continuous after the glycosylation of rapeseed PI with DX. Furthermore, it should be noted that PI-DX 2:1 and PI-DX 1:2 conjugates displayed similar surface morphology, while PI-DX 1:1 conjugate showed an irregular shape with a wrinkled surface and many small particles. This may be because PI-DX 1:1 conjugate acted as a transition state between PI-DX 2:1 and PI-DX 1:2 conjugates.

### 3.5. Functional Properties of PI and PI-DX Conjugates

#### 3.5.1. Solubility

**Figure 4:**
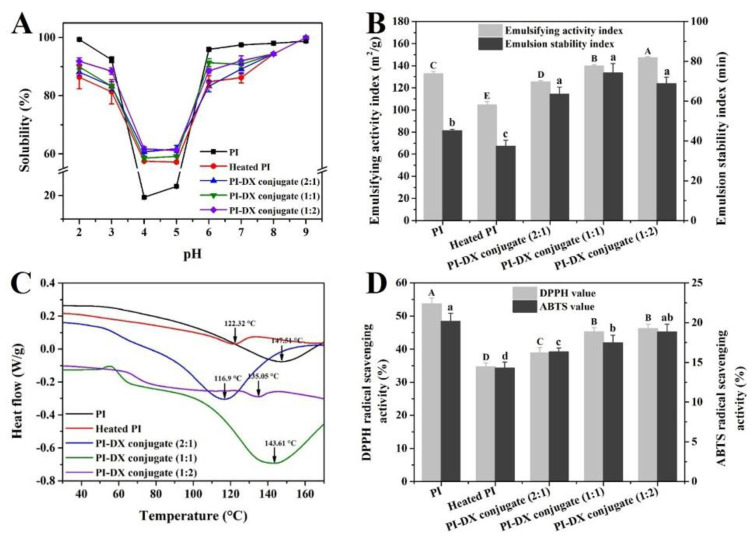
The protein solubility (**A**), emulsifying property (**B**), differential scanning calorimetry curve (**C**), and antioxidant activity (**D**) of PI-DX conjugates. PI: protein isolate; DX: dextran. Values with different letters (A, B, C, D, E, and a, b, c, d) in each sample were significantly different (*p* < 0.05).

The solubility is an important functional property of proteins, which is the first factor to be considered as a raw material in the food industry [12]. As shown in Figure 4A, PI had minimum solubility at pH 4–5, close to the isoelectric point. Compared with PI, heat treatment slightly decreased the solubility at pH 2–3 and 6–8 and significantly increased solubility at pH 4–5. The solubility of the heated PI at the pH 4 and 5 (57.50 and 57.18%, respectively) was higher than that of PI (19.23–23.85%). This may be because PI is involved in the formation of protein aggregates during heating, which remained at pH values between 4 and 5. In addition, the glycosylation could improve the solubility of the heated PI in broad pH values. This may be because the covalent binding of hydrophilic sugars could introduce hydrophilic hydroxyl groups into the proteins [42,43]. This phenomenon became more obvious with the increase in DG [20].

#### 3.5.2. Emulsifying Properties

As a natural emulsifier, plant protein is one of the most important stabilizers used in food emulsion formulation. The stability of a protein emulsion is mainly reflected in the emulsifying activity index (EAI) and emulsion stability index (ESI), which indicate the absorption ability of protein molecules at the oil/water interface [44]. As shown in Figure 4B, both EAI and ESI of PI were decreased by heat treatment. This observation may result from the formation of protein–protein aggregates of PI, leading to a reduction in the ability to form an interfacial film on the surface of the oil droplets [45]. After the Maillard reaction, compared with heated PI, the EAI and ESI of PI-DX conjugates were significantly (*p* < 0.05) improved, even higher than PI. This result may be because the introduction of the DX molecule increased the steric repulsions between protein molecules, thus, improving the emulsifying properties. In addition, the EAI increased gradually with the increase in DX concentration, while the ESI value reached the maximum value in PI-DX 1:1 conjugate. The difference may be because EAI is more sensitive to protein molecular weight, since it relies on rapid adsorption to stabilize the emulsion droplets during homogenization [38]. However, to stabilize the emulsion droplets against recoalescence, a stronger interparticle electrostatic repulsion between protein molecules is required [46]. Therefore, PI-DX 1:2 conjugate with the smallest particle size of the conjugates was considered to be the better emulsifier, and PI-DX 1:1 conjugate had the highest absolute zeta potential value, which was beneficial to the stability of the emulsion.

#### 3.5.3. DSC

DSC is an effective thermal analysis method to determine the thermal behavior of proteins and the interactions between protein–polysaccharides. It is well known that the denaturation peak (T_d_) reflects the temperature at which the protein is denatured and is related to the amino acid composition and spatial conformation of protein, as well as the electrostatic interactions and hydrogen bonds between or within protein molecules [47]. As shown in Figure 4C, the T_d_ values of PI and heated PI were 147.51 and 122.32 °C, respectively, indicating that the thermal stability of PI was reduced upon heating. The change in T_d_ may be due to the conformational change in PI caused by heat treatment, resulting in increased protein–protein interaction and random coil structure content. Compared with heated PI, PI-DX 2:1 conjugate had a slightly lower T_d_ value (116.90 °C), whereas the PI-DX 1:1 and PI-DX 2:1 conjugates had obviously higher T_d_ values (143.61 and 135.05 °C, respectively). These results suggest that the thermal stability of heated PI may be enhanced by the Maillard reaction, especially for the PI-DX 1:1 conjugate. As reported by Robitaille et al. [48], the introduction of polysaccharide molecules increased the steric exclusion and electrostatic repulsion of protein–polysaccharide conjugates, preventing the aggregation of protein at high temperatures. In addition, the higher thermal stability of the PI-DX conjugates can also be reflected by the more ordered structure, which was confirmed by the FTIR results.

#### 3.5.4. Antioxidant Activities

Two different assays were used to evaluate the antioxidant activity of PI and PI-DX conjugates (Figure 4D). The DPPH and ABTS values of PI were 53.66 ± 1.78 and 20.09 ± 1.01%, respectively. However, compared with PI, the DPPH and ABTS values of heated PI were decreased by 35.45 and 29.19%, respectively. The decreased antioxidant activities may be related to the protein oxidation upon heating, resulting in the change in the primary structure of PI, especially amino acid residues [49]. In contrast, the antioxidant activities of heated PI were significantly improved by the Maillard reaction. The highest DPPH and ABTS values were observed in the PI-DX 1:2 conjugate (46.15 ± 1.38 and 18.82 ± 0.99%, respectively). The result indicated the potential of Maillard products as the primary antioxidants. Maillard products prepared at higher temperatures (>80 °C) were reported to exhibit excellent antioxidant activities [37,50]. However, the denaturation of native proteins easily occurred in aqueous solutions during heating at above its denaturation temperature [51]. In this study, the improvement effect of Maillard reaction on PI was achieved by controlling the heating temperature at 60 °C to preserve the original state of PI as much as possible.

## 4. Conclusions

In summary, the results showed that heat treatment had a negative impact on the structure and functionalities of PI, while the properties of PI were significantly improved after the Maillard reaction with DX under wet-heating conditions (60 °C, 5 h). The formation of PI-DX conjugates was confirmed by DG and SDS-PAGE. The FTIR analysis showed that the secondary structure of PI-DX conjugates became more orderly than the heated PI, which was manifested by the reduced random coil content. The increased intrinsic fluorescence and surface hydrophobicity indicated changes in the tertiary structure of PI-DX conjugates. In addition, the PI-DX conjugates exhibited improved functional properties, including solubility, emulsification, thermal stability and antioxidant activities. These results suggested that the Maillard reaction may be a promising approach to enhance the application potentials of PI in food processing.

## Figures and Tables

**Table 1:** Z-average size and zeta potential of the PI and PI-DX conjugates.

Samples	Z-Average Size (nm)	Zeta Potential (mV)
PI	66.33 ± 1.07 ^d^	−21.40 ± 0.70 ^a^
Heated PI	142.90 ± 1.65 ^b^	−23.13 ± 0.61 ^b^
PI-DX conjugate (2:1)	152.90 ± 2.61 ^a^	−23.47 ± 0.42 ^bc^
PI-DX conjugate (1:1)	145.53 ± 0.55 ^b^	−24.63 ± 0.46 ^c^
PI-DX conjugate (1:2)	124.07 ± 1.50 ^c^	−20.63 ± 0.40 ^a^

PI: protein isolate; DX: dextran. Values with different letters (a, b, c, d) in the same column in each sample were significantly different (*p* < 0.05).

**Table 2:** Secondary structure contents of the PI and PI-DX conjugates determined by FTIR.

Samples	α-Helix	β-Sheet	β-Turn	Random Coil
PI	26.51	34.89	17.93	20.66
Heated PI	24.14	30.97	21.96	22.93
PI-DX conjugate (2:1)	24.41	31.59	21.80	22.20
PI-DX conjugate (1:1)	27.72	32.65	18.89	20.74
PI-DX conjugate (1:2)	31.36	34.94	17.08	16.62

## Data Availability

The data presented in this study are available on request from the corresponding author.

## Supplementary Materials

The following supporting information can be downloaded at: https://www.mdpi.com/article/10.3390/foods11193066/s1, Figure S1: The Fourier transform infrared spectroscopy of DX; Figure S2: The Fourier spectrum (upper) and Gaussian fitting curves (bottom) in the amide I region of PI and PI-DX conjugates.
