# Supplementary material for: Dextran Conjugation Improves the Structural and Functional Properties of Heat-Treated Protein Isolate from Cinnamomum camphora Seed Kernel
Source: Foods. 2022 Oct 2;11(19):3066. doi: 10.3390/foods11193066 (PMC9564210; doi:10.3390/foods11193066)
Supplement: Supplementary file 1 [file foods-11-03066-s001.zip › foods-1887016-supplementary.pdf]

Supplementary material

Figure S1

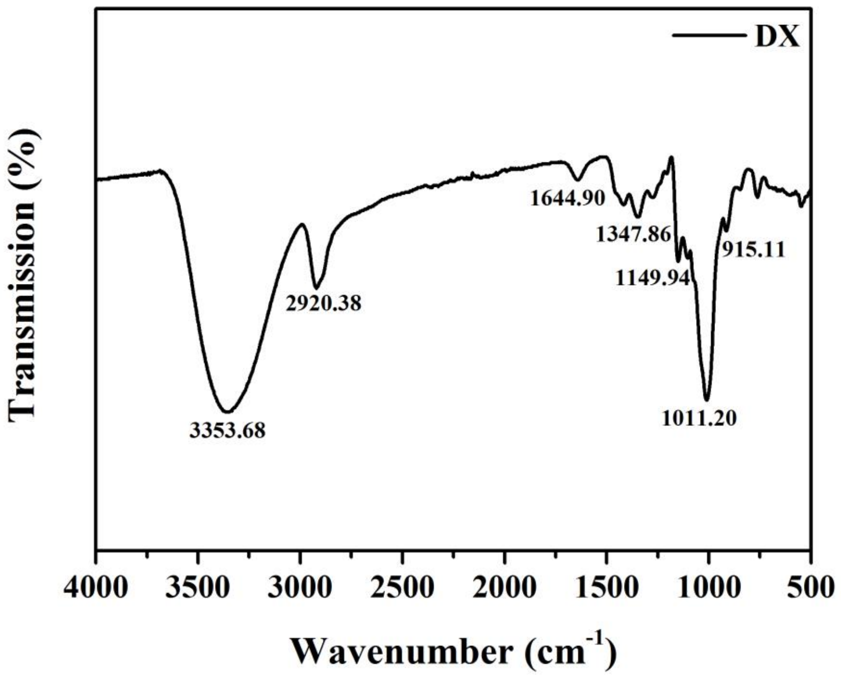

Figure S1. The Fourier transform infrared spectroscopy of DX.

Figure S2

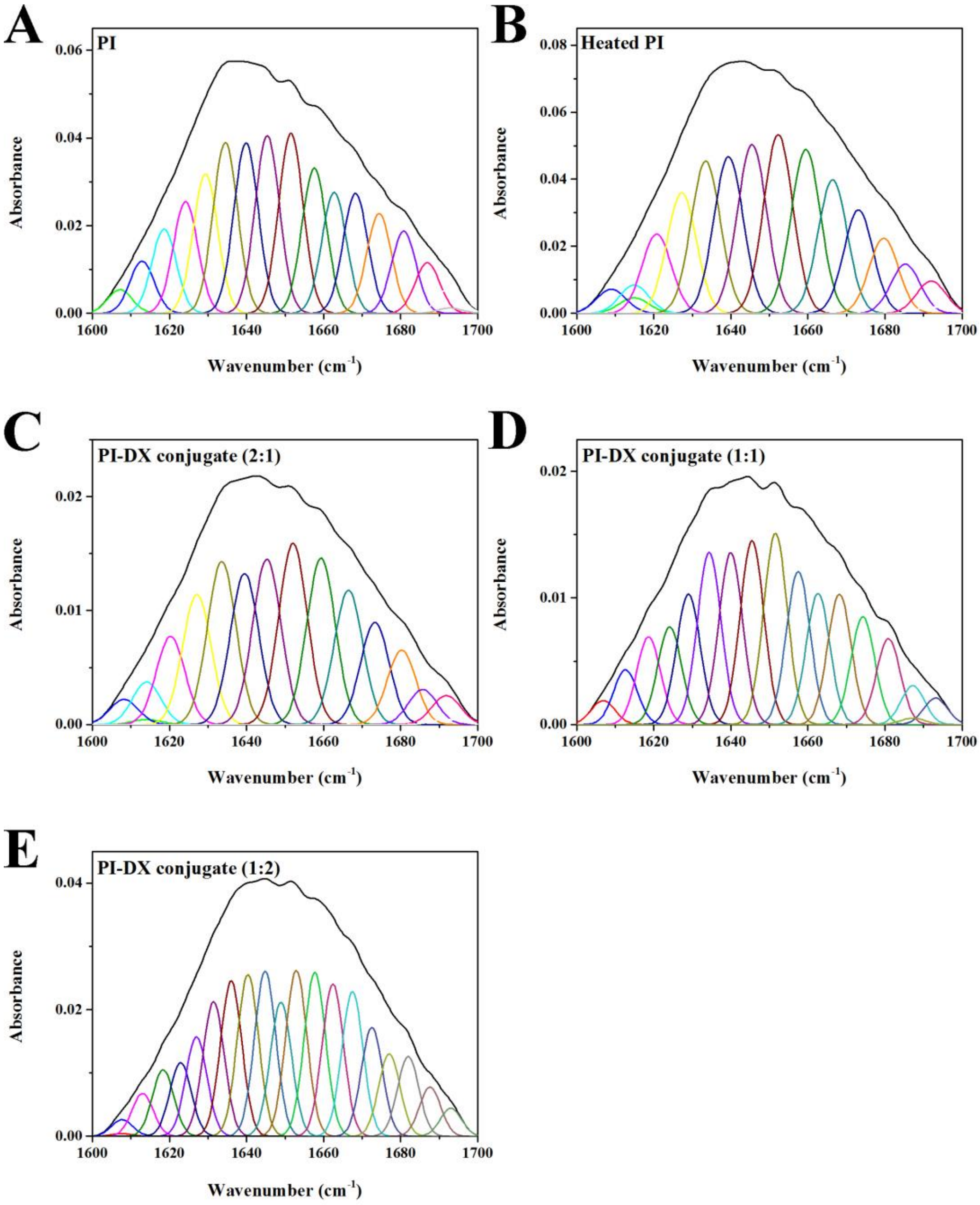

Figure S2. The Fourier spectrum (upper) and Gaussian fitting curves (bottom) in the amide I region of PI and PI−DX conjugates.
